# Supplementary material for: Multi-resource dynamic coordinated planning of flexible distribution network
Source: Nat Commun. 2024 May 29;15:4576. doi: 10.1038/s41467-024-48862-5 (PMC11530552; doi:10.1038/s41467-024-48862-5)
Supplement: Supplementary file 1 — Supplementary Information [file 41467_2024_48862_MOESM1_ESM.pdf]

Supplementary Information

Multi-resource Dynamic Coordinated Planning of Flexible Distribution Network

Rui Wang[1]†, Haoran Ji[1]†, Peng Li[1*], Hao Yu[1*], Jinli Zhao[1], Liang Zhao[2], Yue Zhou[3], Jianzhong Wu[3], Linquan Bai[4], Jinyue Yan[5], Chengshan Wang[1]

1. Key Laboratory of Smart Grid of Ministry of Education, Tianjin University, Tianjin, 300072, China;
2. State Grid Tianjin Electric Power Company, Tianjin 300010, China;
3. School of Engineering, Cardiff University, Cardiff CF24 3AA, UK;
4. Department of Electrical Engineering and Computer Science, University of Tennessee, Knoxville, 37996, USA;
5. School of Sustainable Development of Society and Technology, Mälardalen University, Västerås, 721 23, Sweden

*Corresponding author(s). Peng Li; Hao Yu.

†These authors contributed equally to this work.

Supplementary Tables

Supplementary Table 1. Price associated with planning

Stage	I	II	III	IV
**Transformer investment (10^3CNY/kVA)**	0.5	0.4	0.35	0.3
**Storage battery investment (10^3CNY/kWh)**	1.0	0.8	0.5	0.3
**Converter investment (10^3CNY/kVA)**	0.8	0.6	0.4	0.2
**Land exploitation (10^6CNY)**	3.0	3.5	4.0	5.0
**Line construction (10^6CNY/km)**	0.1	0.12	0.16	0.2
**Electricity purchase (CNY/kWh)**	0.35	0.3	0.25	0.2

Supplementary Figures

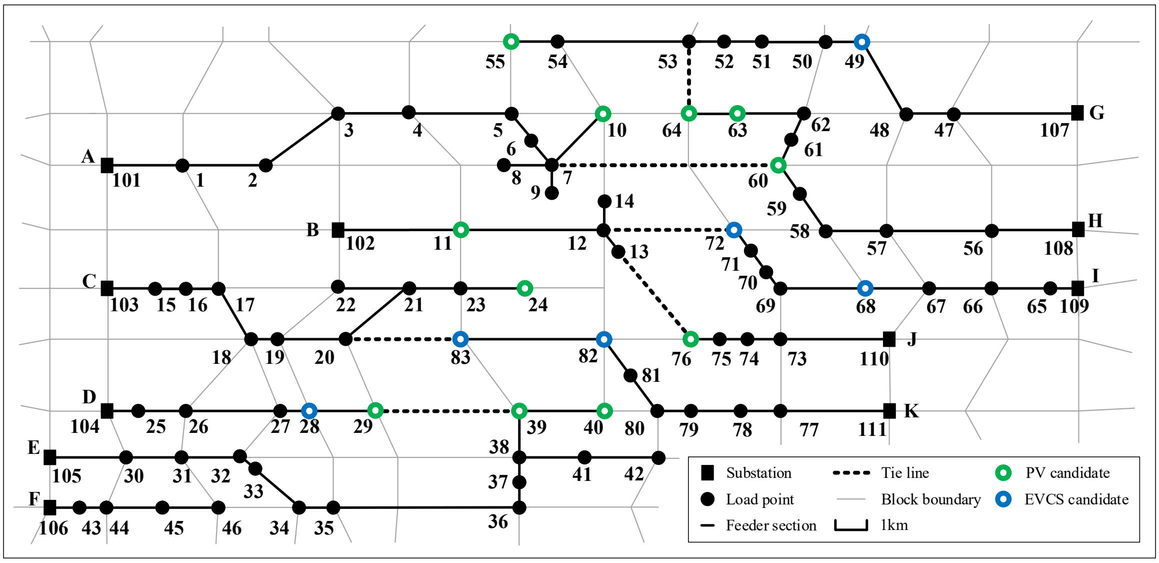

Supplementary Fig. 1. Structure of the practical distribution network. The structure of the distribution network is typically multi-sectioned in design and radial in operation. Candidate nodes for planning EVCS (blue hollow dot) and PV (green hollow dot) can be determined by referring to their geographical, lighting, and economic conditions.

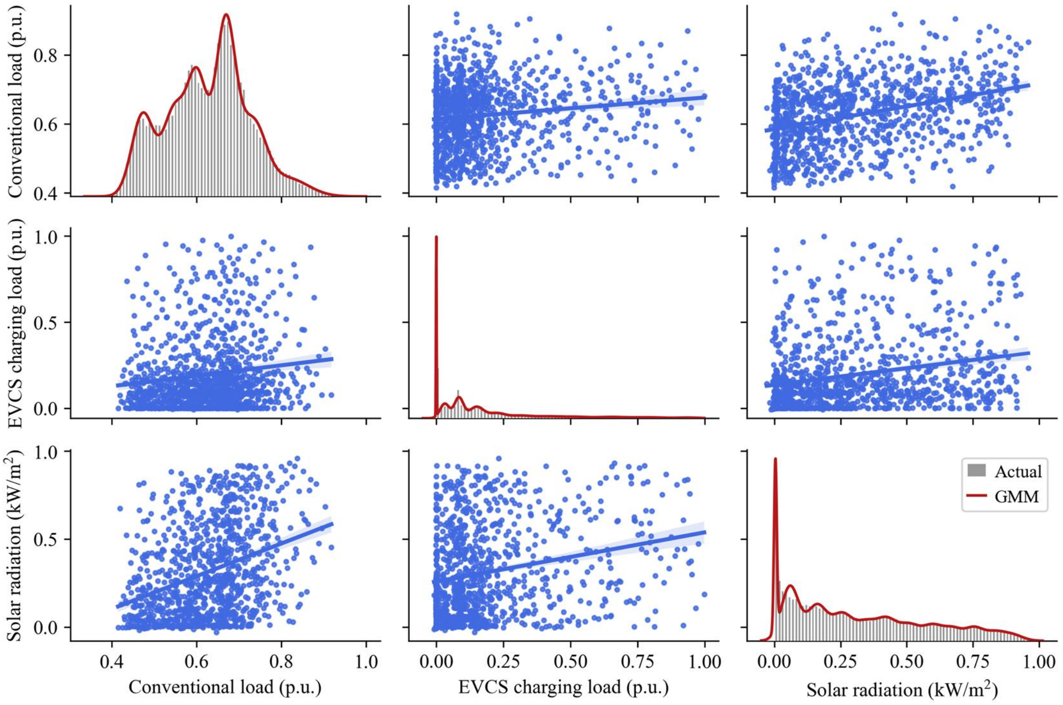

Supplementary Fig. 2. Uncertainty quantification. The univariate probability distributions established by Gaussian mixture models (dark red lines) can effectively reflect the actual probabilistic characteristics (grey histograms) of the sources and loads in FDNs. The pairwise correlations (blue scatters) between different types of uncertainties are handled based on Nataf transformation, as illustrated at the non-diagonal positions of the image.
